# Supplementary material for: Growth Adaptation of gnd and sdhCB Escherichia coli Deletion Strains Diverges From a Similar Initial Perturbation of the Transcriptome
Source: Front Microbiol. 2018 Aug 7;9:1793. doi: 10.3389/fmicb.2018.01793 (PMC6090065; doi:10.3389/fmicb.2018.01793)
Supplement: Supplementary file 2 [file Data_Sheet_2.docx]

Supplementary Materials:

# Title

Growth adaptation of *gnd* and *sdhCB Escherichia coli* deletion strains diverges from a similar initial perturbation of the transcriptome

# Authors

Douglas McCloskey^1,2^, Sibei Xu^1^, Troy E. Sandberg^1^, Elizabeth Brunk^1^, Ying Hefner^1^, Richard Szubin^1^, Adam M. Feist^1,2,^ , and Bernhard O. Palsson^1,2,^*

# Affiliations

^1^Department of Bioengineering, University of California - San Diego, La Jolla, CA 92093, USA.

^2^Novo Nordisk Foundation Center for Biosustainability, Technical University of Denmark, 2800 Lyngby, Denmark.

*Corresponding author, Department of Bioengineering, University of California, San Diego, 9500 Gilman Drive, La Jolla, CA 92093-0412, USA.

Tel.: [858-534-5668]; Fax: [858-822-3120]; E-mail: [bpalsson@ucsd.edu](mailto:bpalsson@ucsd.edu)

# Table of Contents

[**Title**](#_361ha6xzm7sx) **1**

[**Authors**](#_ejm29frs2kgr) **1**

[**Affiliations**](#_uqn5zqxz25r0) **1**

[**Table of Contents**](#_1v34vj3rjem5) **2**

[**Supplementary Figures**](#_5axprxg37e2z) **2**

[Fig. S1](#_li5usmquohx5) 2

[Fig. S2](#_ckdrq728qx4) 3

[**Supplemental Tables:**](#_xt9wo8vjf77y) **4**

[Table S1:](#_cmb6m31om6kt) 4

[Table S2:](#_urygh6fxa7m4) 4

[Table S3:](#_yb6px5jjsr3n) 4

[Table S4:](#_kdn7bbnc99i4) 4

[Table S5:](#_jdov1s708se2) 4

[Table S6:](#_oo1h5je2vfg1) 5

[Table S7:](#_gmg2483seub6) 5

[Table S8:](#_g8uynsxlsu4c) 5

[**References**](#_2ng3iu7uf9hw) **6**

#

# Supplementary Figures

## Fig. S1

Gain of function mutations in eGnd strains that relieved cycling of isoleucine biosynthesis. A) Operon schematic of the *ilv* operon, which encodes genes involved in isoleucine biosynthesis. In *E. coli* K-12 strains, an internal frameshift mutation cuts the *ilvG* gene into two non-functional segments [(Favre et al. 1976)](https://paperpile.com/c/7Obq0e/0MlB), which leads to oscillations in isoleucine biosynthesis [(Andersen et al. 2001)](https://paperpile.com/c/7Obq0e/ljV1). A removal of a single nucleotide or addition of two nucleotides can restore *ilvG* expression [(Lawther et al. 1981, 1982)](https://paperpile.com/c/7Obq0e/HyUp+u5qQ). B) Mutation frequency and expression levels of genes involved in isoleucine biosynthesis. Note that single nucleotide deletion mutations (DEL) in eGnd01 and eGnd03 were found that restored *ilvG* expression.

## Fig. S2

Gene expression perturbations in sulfur metabolism in eSdhCB strains. A) Network schematic of the sulfur metabolic pathways. The sulfur metabolic pathway converts sulfate (so4), asp-L, L-serine (ser-L) and Succinyl-CoA (succoa) to L-cysteine (cys-L), which is then converted to L-methionine (met-L). B) Gene expression and metabolic flux level for eSdhCB strains.

# Supplemental Tables:

## Table S1:

List of primers used to generate the KO strains in this study

## Table S2:

Growth rates, substrate uptake and secretion rates of the initial knockout strains and evolved endpoints.

## Table S3:

Absolute metabolite concentrations for all ref, uKO, and eKO strains in the study

## Table S4:

Gene expression differences for all ref, uKO, and eKO strains in the study

## Table S5:

Gene expression normalized counts for all ref, uKO, and eKO strains in the study

## Table S6:

Absolute metabolic flux values for all ref, uKO, and eKO strains in the study

## Table S7:

Metabolic model used for MFA and sampling simulations

## Table S8:

Annotated mutations

# References

1. [LaCroix, R. A. *et al.* Use of Adaptive Laboratory Evolution To Discover Key Mutations Enabling Rapid Growth of Escherichia coli K-12 MG1655 on Glucose Minimal Medium. *Appl. Environ. Microbiol.* **81,** 17–30 (2015).](http://paperpile.com/b/evXh0y/33ont)

2. [Sandberg, T. E. *et al.* Evolution of Escherichia coli to 42 °C and subsequent genetic engineering reveals adaptive mechanisms and novel mutations. *Mol. Biol. Evol.* **31,** 2647–2662 (2014).](http://paperpile.com/b/evXh0y/qnJae)

3. [Datsenko, K. A. & Wanner, B. L. One-step inactivation of chromosomal genes in Escherichia coli K-12 using PCR products. *Proc. Natl. Acad. Sci. U. S. A.* **97,** 6640–6645 (2000).](http://paperpile.com/b/evXh0y/XNFqU)

4. [Sambrook, J. & Russell, D. W. Molecular cloning: a laboratory manual 3rd edition. *Coldspring-Harbour Laboratory Press, UK* (2001).](http://paperpile.com/b/evXh0y/AKa9i)

5. [Fong, S. S. *et al.* In silico design and adaptive evolution of Escherichia coli for production of lactic acid. *Biotechnol. Bioeng.* **91,** 643–648 (2005).](http://paperpile.com/b/evXh0y/jqUBe)

6. [Orth, J. D. *et al.* A comprehensive genome-scale reconstruction of Escherichia coli metabolism--2011. *Mol. Syst. Biol.* **7,** 535 (2011).](http://paperpile.com/b/evXh0y/fy7oz)

7. [Schellenberger, J. & Palsson, B. Ø. Use of randomized sampling for analysis of metabolic networks. *J. Biol. Chem.* **284,** 5457–5461 (2009).](http://paperpile.com/b/evXh0y/hW5XH)

8. [McCloskey, D., Gangoiti, J. A., Palsson, B. O. & Feist, A. M. A pH and solvent optimized reverse-phase ion-paring-LC–MS/MS method that leverages multiple scan-types for targeted absolute quantification of intracellular metabolites. *Metabolomics* **11,** 1338–1350 (2015).](http://paperpile.com/b/evXh0y/cxP4T)

9. [McCloskey, D., Young, J. D., Xu, S., Palsson, B. O. & Feist, A. M. MID Max: LC-MS/MS Method for Measuring the Precursor and Product Mass Isotopomer Distributions of Metabolic Intermediates and Cofactors for Metabolic Flux Analysis Applications. *Anal. Chem.* **88,** 1362–1370 (2016).](http://paperpile.com/b/evXh0y/oCOk1)

10. [McCloskey, D. *et al.* A model-driven quantitative metabolomics analysis of aerobic and anaerobic metabolism in E. coli K-12 MG1655 that is biochemically and thermodynamically consistent. *Biotechnol. Bioeng.* **111,** 803–815 (2014).](http://paperpile.com/b/evXh0y/Zl2y1)

11. [McCloskey, D., Utrilla, J., Naviaux, R. K., Palsson, B. O. & Feist, A. M. Fast Swinnex filtration (FSF): a fast and robust sampling and extraction method suitable for metabolomics analysis of cultures grown in complex media. *Metabolomics* **11,** 198–209 (2014).](http://paperpile.com/b/evXh0y/axTv2)

12. [Honaker, J., King, G. & Blackwell, M. Amelia II: A Program for Missing Data. *J. Stat. Softw.* **45,** 1–47 (2011).](http://paperpile.com/b/evXh0y/7ExOh)

13. [Rocke, D., Tillinghast, J., Durbin-Johnson, B. & Wu, S. L. LMGene Software for Data Transformation and Identification of Differentially Expressed Genes in Gene Expression Arrays. R package version 2.4. 0.](http://paperpile.com/b/evXh0y/CFzyG)

14. [Young, J. D. INCA: a computational platform for isotopically non-stationary metabolic flux analysis. *Bioinformatics* **30,** 1333–1335 (2014).](http://paperpile.com/b/evXh0y/CaVnj)

15. [McCloskey, D., Young, J. D., Xu, S., Palsson, B. O. & Feist, A. M. Modeling Method for Increased Precision and Scope of Directly Measurable Fluxes at a Genome-Scale. *Anal. Chem.* **88,** 3844–3852 (2016).](http://paperpile.com/b/evXh0y/ESuhj)

16. [Megchelenbrink, W., Huynen, M. & Marchiori, E. <italic>optGpSampler</italic>: An Improved Tool for Uniformly Sampling the Solution-Space of Genome-Scale Metabolic Networks. *PLoS One* **9,** e86587 (2014).](http://paperpile.com/b/evXh0y/z9ZLF)

17. [Langmead, B., Trapnell, C., Pop, M. & Salzberg, S. L. Bowtie: an ultrafast memory-efficient short read aligner. *Genome Biol.* **10,** R25 (2009).](http://paperpile.com/b/evXh0y/nEyvG)

18. [Trapnell, C. *et al.* Transcript assembly and quantification by RNA-Seq reveals unannotated transcripts and isoform switching during cell differentiation. *Nat. Biotechnol.* **28,** 511–515 (2010).](http://paperpile.com/b/evXh0y/MxNvn)

19. [Deatherage, D. E. & Barrick, J. E. Identification of mutations in laboratory-evolved microbes from next-generation sequencing data using breseq. *Methods Mol. Biol.* **1151,** 165–188 (2014).](http://paperpile.com/b/evXh0y/22LuW)

20. [Berman, H. M. *et al.* The Protein Data Bank. *Nucleic Acids Res.* **28,** 235–242 (2000).](http://paperpile.com/b/evXh0y/j97vU)

21. [Berman, H., Henrick, K. & Nakamura, H. Announcing the worldwide Protein Data Bank. *Nat. Struct. Biol.* **10,** 980 (2003).](http://paperpile.com/b/evXh0y/EL0mA)

22. [Xu, D. & Zhang, Y. Ab Initio structure prediction for Escherichia coli: towards genome-wide protein structure modeling and fold assignment. *Sci. Rep.* **3,** 1895 (2013).](http://paperpile.com/b/evXh0y/ym2uT)

23. [Wu, S., Skolnick, J. & Zhang, Y. Ab initio modeling of small proteins by iterative TASSER simulations. *BMC Biol.* **5,** 17 (2007).](http://paperpile.com/b/evXh0y/aQFOT)

24. [Keseler, I. M. *et al.* EcoCyc: fusing model organism databases with systems biology. *Nucleic Acids Res.* **41,** D605–12 (2013).](http://paperpile.com/b/evXh0y/eZEwG)

25. [Humphrey, W., Dalke, A. & Schulten, K. VMD: visual molecular dynamics. *J. Mol. Graph.* **14,** 33–8, 27–8 (1996).](http://paperpile.com/b/evXh0y/R2UcR)

26. [Nyström, T. The glucose-starvation stimulon of Escherichia coli: induced and repressed synthesis of enzymes of central metabolic pathways and role of acetyl phosphate in gene expression and starvation survival. *Mol. Microbiol.* **12,** 833–843 (1994).](http://paperpile.com/b/evXh0y/6yUTL)

27. [Hesslinger, C., Fairhurst, S. A. & Sawers, G. Novel keto acid formate-lyase and propionate kinase enzymes are components of an anaerobic pathway in Escherichia coli that degrades L-threonine to propionate. *Mol. Microbiol.* **27,** 477–492 (1998).](http://paperpile.com/b/evXh0y/VD8iw)

28. [Majdalani, N. & Gottesman, S. The Rcs phosphorelay: a complex signal transduction system. *Annu. Rev. Microbiol.* **59,** 379–405 (2005).](http://paperpile.com/b/evXh0y/JOsQ0)

29. [Hommais, F. *et al.* GadE (YhiE): a novel activator involved in the response to acid environment in Escherichia coli. *Microbiology* **150,** 61–72 (2004).](http://paperpile.com/b/evXh0y/7PiGo)

30. [Cho, Y. *et al.* Individual and collective contributions of chaperoning and degradation to protein homeostasis in E. coli. *Cell Rep.* **11,** 321–333 (2015).](http://paperpile.com/b/evXh0y/hMVAv)

31. [Wohlever, M. L., Baker, T. A. & Sauer, R. T. Roles of the N domain of the AAA+ Lon protease in substrate recognition, allosteric regulation and chaperone activity. *Mol. Microbiol.* **91,** 66–78 (2014).](http://paperpile.com/b/evXh0y/5XNdB)

32. [Meenakshi, S. & Munavar, M. H. Suppression of capsule expression in Δlon strains of Escherichia coli by two novel rpoB mutations in concert with HNS: possible role for DNA bending at rcsA promoter. *Microbiologyopen* **4,** 712–729 (2015).](http://paperpile.com/b/evXh0y/ikxBd)

33. [Ebel, W. & Trempy, J. E. Escherichia coli RcsA, a positive activator of colanic acid capsular polysaccharide synthesis, functions To activate its own expression. *J. Bacteriol.* **181,** 577–584 (1999).](http://paperpile.com/b/evXh0y/gapMA)

34. [Gervais, F. G., Phoenix, P. & Drapeau, G. R. The rcsB gene, a positive regulator of colanic acid biosynthesis in Escherichia coli, is also an activator of ftsZ expression. *J. Bacteriol.* **174,** 3964–3971 (1992).](http://paperpile.com/b/evXh0y/Ltuhk)

35. [Wehland, M. & Bernhard, F. The RcsAB box. Characterization of a new operator essential for the regulation of exopolysaccharide biosynthesis in enteric bacteria. *J. Biol. Chem.* **275,** 7013–7020 (2000).](http://paperpile.com/b/evXh0y/VwXnZ)

36. [Francez-Charlot, A. *et al.* RcsCDB His-Asp phosphorelay system negatively regulates the flhDC operon in Escherichia coli. *Mol. Microbiol.* **49,** 823–832 (2003).](http://paperpile.com/b/evXh0y/UelV0)

37. [Ferrières, L., Aslam, S. N., Cooper, R. M. & Clarke, D. J. The yjbEFGH locus in Escherichia coli K-12 is an operon encoding proteins involved in exopolysaccharide production. *Microbiology* **153,** 1070–1080 (2007).](http://paperpile.com/b/evXh0y/KqKXF)
