# Supplementary figures and images for: Growth Adaptation of gnd and sdhCB Escherichia coli Deletion Strains Diverges From a Similar Initial Perturbation of the Transcriptome
Source: Front Microbiol. 2018 Aug 7;9:1793. doi: 10.3389/fmicb.2018.01793 (PMC6090065; doi:10.3389/fmicb.2018.01793)

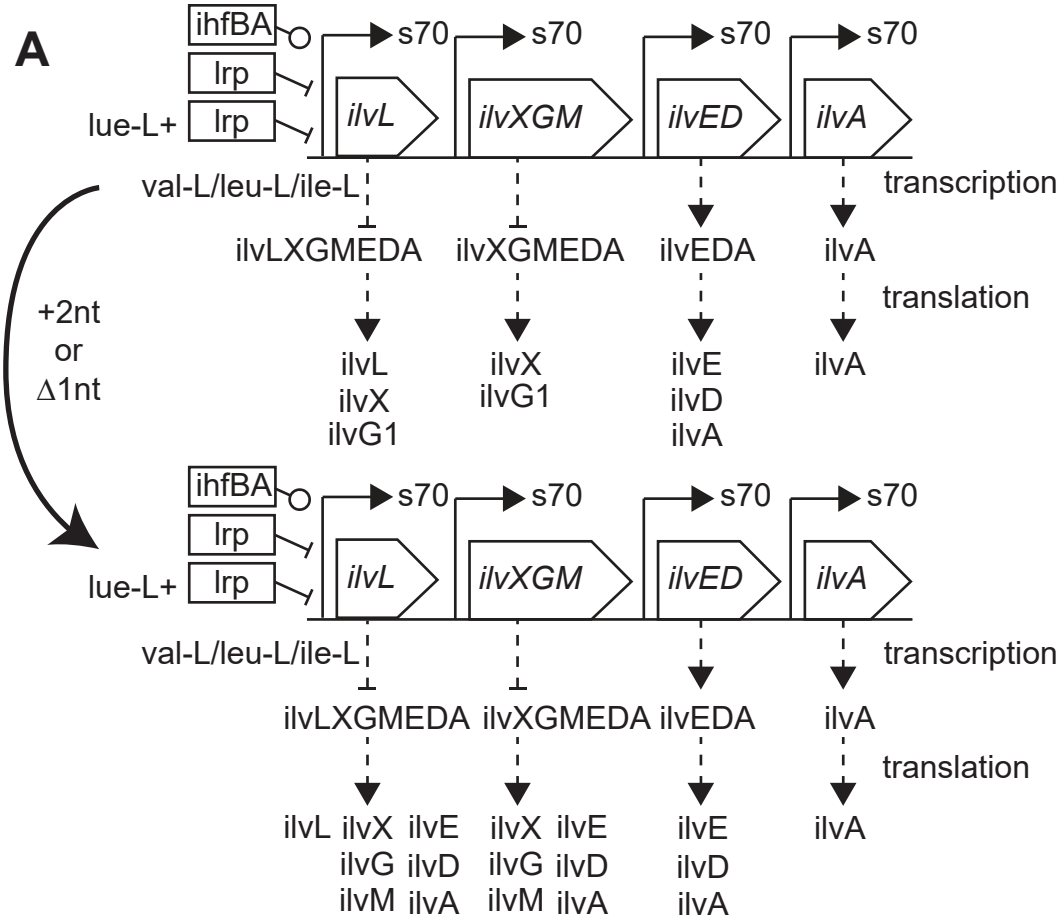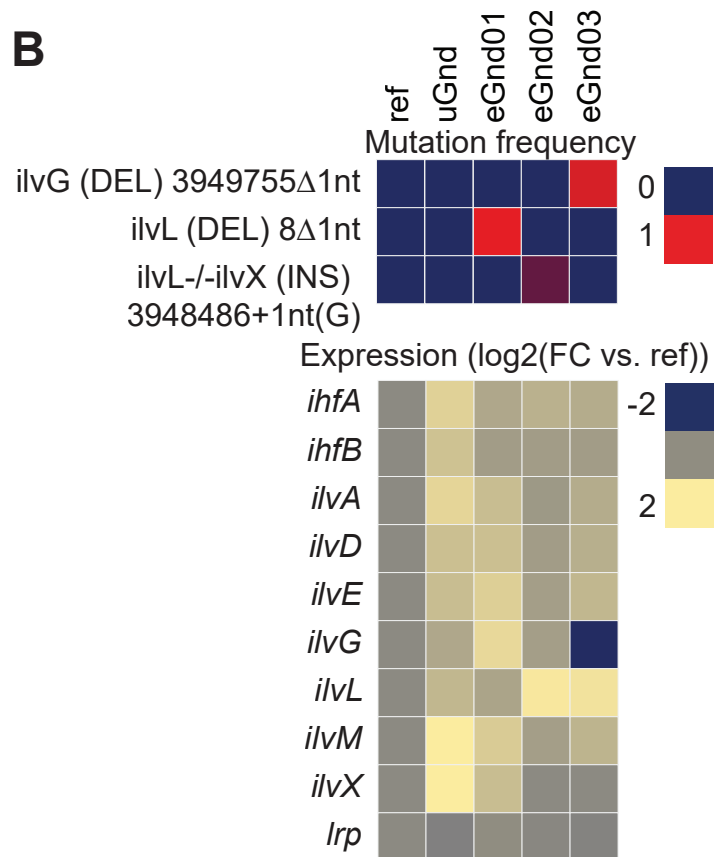

Supplement: Supplementary file 3 [file Image_1.PDF]

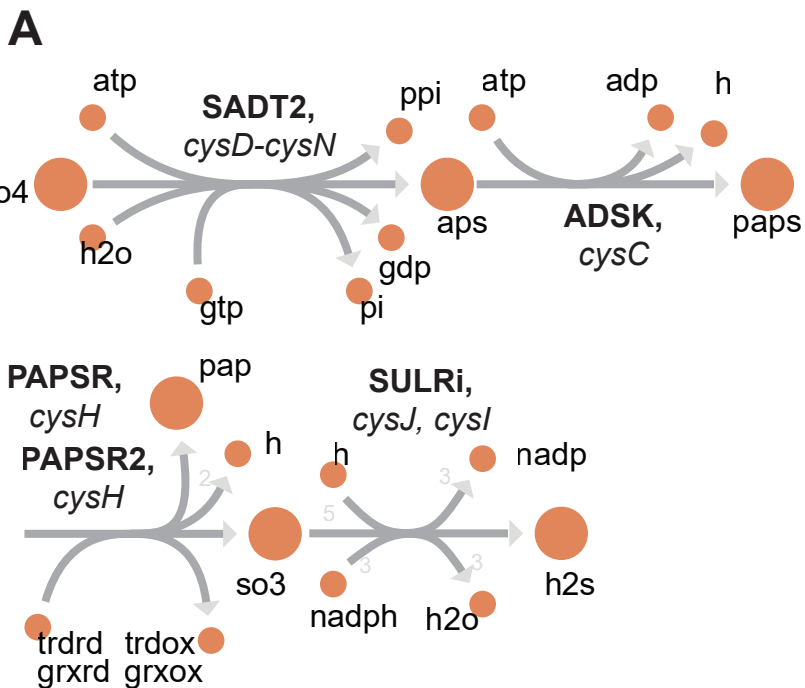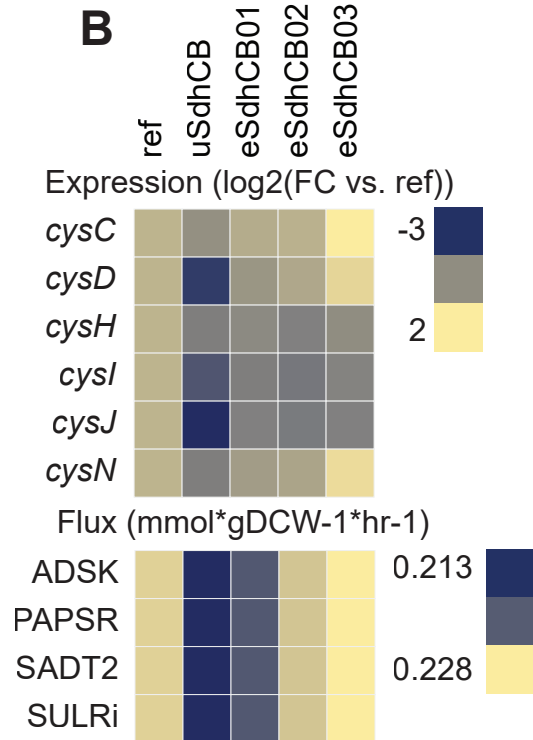

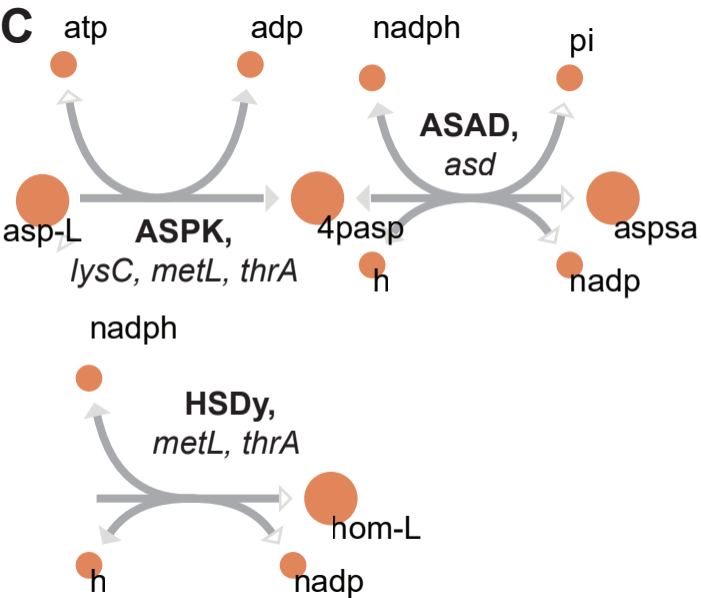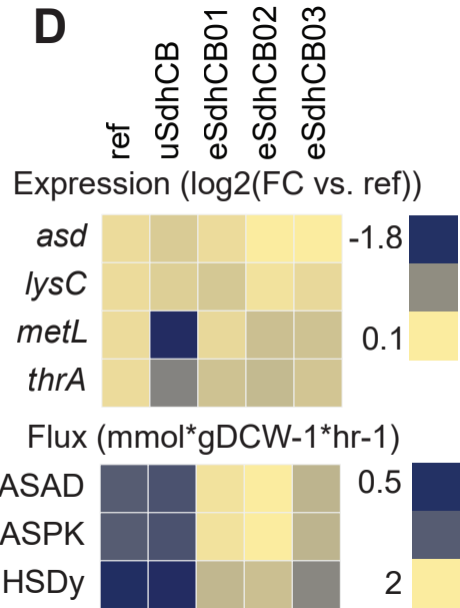

Supplement: Supplementary file 4 [file Image_2.PDF]
